# The Medical Association of Central New York

**Published:** 1897-12

**Authors:** C. A. Vander Beek


### Society Proceedings.

### THE MEDICAL ASSOCIATION OF CENTRAL NEW YORK.

Reported by C. A. VANDER BEEK, Secretary.

THE Thirtieth Annual Meeting was held at the Ellicott Club, Buffalo, N.Y., Tuesday, October 19, 1897. The President, Dr. Edward B. Angell, of Rochester, called the meeting to order at 10.25 o'clock.

Upon motion of Dr. Gerin the reading of the minutes of the previous meeting was dispensed with.

The PRESIDENT appointed the following committees :

*Credentials.*—Drs. E. L. Mooney, Onondaga ; B. C. Loveland, Ontario ; John Gerin, Cayuga.

*Business.*—Drs. Lucien Howe, Erie ; John O. Roe, Monroe ; J. P. Creveling, Cayuga.

*Reception.*—Drs. W. W. Potter, C. G. Stockton, Wm. C. Krauss, Edw. Clark, Franklin C. Gram, Erie.

*Publication.*—Drs. Wm. C. Krauss, Erie ; C. A. Van der Beek, S. L. Elsner, Monroe.

The report of the treasurer showed :

| | |
|---|---|
| Receipts | $288.77 |
| Disbursements | 124.90 |
| Balance on hand | $163.87 |

Upon motion of Dr. GERIN the report was accepted.

On request of the President, the Vice-president was called upon to preside.

Vice-president Dr. KRAUSS took the chair and said : Before listening to the address of the President I will say that the Association is the guest of the Medical Society of the County of Erie, and also of the Ellicott Club. Everything that we have is yours. Take all you can and be sure and come back again three years from now with a good remembrance of the time you have had here today.

In calling for the address of the President, I don't believe Dr. ANGELL needs any introduction, as he is well known to us through his many years of service as secretary of this Association, and I am sure we will listen to his address with a great deal of pleasure, profit and entertainment. (Applause.)

The President, Dr. EDWARD B. ANGELL, of Rochester, delivered the annual address, entitled, The hygiene of the mind.

Upon motion of Dr. CREVELING a vote of thanks was tendered the President for his most excellent address.

The PRESIDENT then resumed the chair.

The following members and delegates registered :

*Cayuga.*

| | | |
|---|---|---|
| Cheesman, Wm. S. | Foreman, E. S. | Lewis, LeRoy |
| Creveling, J. P. | Gerin, Jno. | Sefton, F. |

*Chautauqua.*

Evarts, R. M.

*Cortland.*

Higgins, E. W.

*Erie.*

Andrews, C. H.
Bayliss, A. W.
Benedict, A. L.
Burnham, N. L.
Clark, Edward
Clemesha, J. C.
Cohen, B.
Colton, A. J.
Congdon, C. E.
Crego, F. S.
Dowd, J. Henry
Dunham, S. A.
Frederick, C. C.
Goltra, J. N.
Gram, F. C.
Grosvenor, J. W.
Hoyer, F. F.
Hubbell, A. A.
Krauss, W. C.
Mann, M. D.
McGuire, F. W.
Millener, F. H.
Mulford, H. J.
Mynter, H.
Porter, J. S.
Reynolds, C. J.
Rochester, DeLancy
Rogers, B. F.
Smith, E. A.
Stockton, C. G.
Thoma, Fridolin
Ullman, Julius
Walsh, J. J.
Wende, G. W.

*Genesee.*

Andrews, L. B.
Stone, F. L.
Whittier, E.

*Livingston.*

Perry, E. C.

*Monroe.*

Brown, W. M.
Cook, R. G.
Culkin, J. R.
Davis, J. C.
Dolley, Sarah R. A.
Earl, E. H.
Elsner, S. L.
Ely, W. S.
Finnessy, Jas. H.
Flick, J. W.
Graham, H. C. W.
Henckell, A. W.
Herriman, W. J.
Howe, Wm. J.
Howell, H. B.
Howk, L. W.
Huber, C. A.
Jones, F. A.
Jones, O. E.
La Moure, C. T.
Lane, G. A.
Leary, M. E.
Maloney, F. W.
McCauley, J. W.
Mooney, Thos. T.
Mulligan, W. T.
Puffer, S. W.
Roe, Jno. O.
Rose, L. W.
Sawers, F. H.
Slaight, Mary J.
Vander Beek, C. A.
Weigel, L. A.
Wolff, W. D.

*Niagara.*

Falkner, W. J.

*Onondaga.*

Clark, G. E.
Cone, Allen.
Cullings, Helen M.
Elsner, H. L.
Hawley, H. B.
Jacobson, N.
Kellogg, W. C.
Mooney, E. L.
Marlow, F. W.
Snow, S. F.
Stephenson, F. H.
Vadeboncœur, A. F.

*Ontario.*

Eiseline, S. A.
Hallenbeck, O. J.
Jackson, C. O.
Loveland, B. C.
Lichty, Jno. A.
Lichty, Cora L.
Mead, A. M.
Thayer, C. C.

*Orleans.*

Caverly, M. L.
Dugan, Jno.
Gould, F. B.
Storer, F. B.

*Oswego.*

Crispell, E. W.

*Steuben.*

Kelly, J. G.

*Wayne.*

Brownell, M. Alice
Young, A. A.

*Wyoming.*

Goodwin, P. S.
King, W. E.
Straight, A. B.

Upon motion the following visitors were invited to be guests of the society, and to take part in the discussions :

VISITORS.

E. Whittier, Genesee county ; Edward Clark, Fridolin Thoma, Franklin C. Gram, N. L. Burnham, Jas. S. Porter, John J. Walsh, J. N. Goltra, Chas. E. Congdon, C. H. Andrews, M. D. Mann, Erie ; S. W. Puffer, Mr. John Dennis, J. R. Culkin, Mary J. Slaight, Monroe county ; W. J. Falkner, Niagara county.

Dr. Howe, chairman of the business committee, announced that papers would be limited to ten minutes and discussions to five minutes, at the end of eight minutes a warning would be given, and at the end of ten minutes final notice by a tap of the bell.

Papers were then read as follows : A case of unusual defective development in an infant—consisting of compound complicated hare-lip, and bilateral maxillary fissure, together with attachment of the intermaxillary bone to the end of the nose. Correction of this deformity by plastic operations. By John O. Roe, M. D., Rochester.

Report of a case with exhibition of the patient. By Charles G. Stockton, M. D., Buffalo.

Report of special committee on remedial legislation in regard to expert testimony. By Frederick Sefton, M. D., chairman.

Upon motion of Dr. H. L. Elsner all discussion and action upon this report was postponed until the papers of Dr. Henckell and Mr. Tracy C. Becker had been read.

Surgical treatment of tubercular peritonitis. By J. P. Creveling, M. D., Auburn.

Discussed by Drs. Nathan Jacobson, Syracuse; W. S. Cheesman, Auburn; John A. Lichty, Clifton Springs; M. D. Mann, Buffalo; H. L. Elsner, Syracuse, and in conclusion by Dr. Creveling.

The value of expert testimony in medico-legal cases. From the medical standpoint, by A. W. Henckell, M. D., Rochester. From the legal standpoint, by Tracy C. Becker, Esq., Buffalo.

The use of the fluorometer in Röntgen-ray work. By Mr. John Dennis, Rochester.

The society then adjourned for luncheon, to re-convene at 2 o'clock.

The President on behalf of the Medical Society of the County of Erie then invited all the members of the Association, the delegates and registered visitors to a luncheon, which was served in the private dining rooms of the Club.

The President called the Society to order at 2.30 p. m.

Dr. Gerin announced the names of the following gentlemen who had complied with the constitution and by-laws and moved their election to permanent membership: Drs. F. W. Higgins, Cortland county; F. B. Storer, M. L. Caverly, F. B. Gould, Orleans county; J. W. McCauley, T. T. Mooney, F. A. Jones, L. W. Howk, R. G. Cook, Jas. H. Finnessy, H. C. W. Graham, C. T. LaMoure, C. A. Huber, Monroe county; A. F. Vadeboncœur, Allen Cone, Onondaga county; J. A. Lichty, C. C. Thayer, Ontario county; Frederick Sefton, Cayuga county; J. Henry Dowd, Erie county; A. B. Straight, Wyoming county. They were so elected.

Dr. Gerin: For a great number of years Auburn has been sending a large delegation to the meetings of this society, and has accepted of the very kind treatment of the different cities, and we are anxious now, sir, to do our part in carrying on the work of the Association. I therefore offer the following amendment to Article II. of the constitution, and move its adoption.

Article II. of the constitution shall hereafter read as follows:

The Association shall meet annually on the third Tuesday of October, the meetings to be held alternately in Rochester, Buffalo, Syracuse and Auburn.

The motion was seconded by Dr. Creveling, of Cayuga. After discussion by the President and Drs. Gerin and Creveling the amendment was adopted.

A doubt having risen as to whether or not notice of this amendment was given at the meeting of 1896, the chair ruled—upon the affirmative assertion of Dr. Gerin—that, inasmuch as the stenographic notes of said meeting were incomplete he would hold that notice had been given in accordance with Article XVII.

The election of officers for the ensuing year now being in order, the chair appointed as tellers: Drs. Young, of Wayne, and Maloney, of Monroe, and announced that a formal ballot would be taken.

Officers elected for the ensuing year are: President, Dr. Wm. C. Krauss, of Buffalo; first vice-president, Dr. E. L. Mooney, of Syracuse; second vice-president, Dr. E. S. Foreman, of Auburn. The secretary and treasurer continue in office another year.

Dr. Gerin then read the following letter, which was afterward ordered printed in the Transactions:

343 Sherbourne Street,

Toronto, October 18, 1897.

The President *New York Central Medical Association.*

*My Dear Mr. President*—The eighth annual meeting of The American Electro-Therapeutic Association will be held in Buffalo, on the second Tuesday in September, 1898, and two following days and we should be very much pleased to receive a delegate from your association.

We also extend a most cordial invitation to your members to attend our sessions.

Our secretary, Dr. John Gerin, of Auburn, N. Y., will present this.

Fraternally yours,

CHARLES R. DICKSON,
*President A. E. T. A.*

Upon motion, the President was designated the delegate to represent this association at the meeting of the American Electro-Therapeutic Association, which meets in Buffalo next year.

The President then announced that a motion would be entertained authorising the secretary to issue credentials to delegates from this society to the meeting of the American Medical Association. The notice was not responded to.

While the tellers were preparing the ballots for president, the paper entitled, A rare case of sarcoma cutis, with exhibition of patient, by Grover W. Wende, M. D., Buffalo, was called for and read.

The President here called for the report of the committee on remedial legislation, of which Dr. Sefton is chairman.

Dr. SEFTON, chairman of said committee, reported as follows: After further consideration of the matter in the committee and with different members of this society, there has been drafted a resolution that will be introduced for your action, viz.:

*Resolved,* That this report (referring to the report of the special committee) be received and the committee be continued and be empowered to confer with committees of the different state medical societies upon remedial legislation with regard to expert medical testimony, and if possible to prepare a bill to be presented at the coming session of the legislature.

Dr. HOWE moved the adoption of the resolution, which was seconded by Dr. Gerin and carried.

Upon motion of Dr. JACOBSON it was ordered that a reprint of the various matters pertaining to medical expert testimony, which have been presented before this society today, be published in pamphlet form for distribution as the committee (on remedial legislation in regard to expert testimony) may desire.

After full discussion regarding the publication of the transactions of the society, and the offer by Dr. Krauss, on behalf of the BUFFALO MEDICAL JOURNAL, to furnish 200 copies for $50, the society voted an appropriation of said sum for the purposes named.

The reading of papers being next in order, Dr. HOWE, of the business committee, reports as follows:

I think it is hardly necessary to apologise to those from Buffalo who have prepared papers, that their papers will be placed last in the list, or after having heard at least from those out of town.

The next to be called for is the one entitled, The Adirondacks in winter for tubercular patients, by SARGENT F. SNOW, M. D., Syracuse.

Dr. STEPHENSON, the second vice-president, then took the chair, after which the paper was discussed by Drs. Mooney, of Syracuse; Rochester, of Buffalo; Ely, of Rochester; Elsner, of Syracuse; Loveland, of Clifton Springs, and in conclusion by Dr. Snow.

The treatment of congenital dislocation of the hip by the Lorenz Method, radiograph illustrations, by L. A. WEIGEL, M. D., Rochester.

Discussed by Dr. Mynter, of Buffalo, and in conclusion by Dr. Weigel.

A case of appendicitis with unusual sequelæ, by NATHAN JACOBSON, M. D., Syracuse,

Clinical reports with specimens. By HENRY L. ELSNER, M.D., Syracuse.

Discussed by Drs. Rochester and Benedict, of Buffalo, and in conclusion by Dr. Elsner.

Report of a case of Jacksonian epilepsy. By F. H. STEPHENSON, M. D., Syracuse.

Some popular errors in the treatment of nervous prostration. By B. C. LOVELAND, M. D., Clifton Springs.

Discussed by Drs. Mann, of Buffalo; Angell and Weigel, of Rochester.

Carcinoma of the testicle. By A. A. YOUNG, M. D., Newark.

The uses of the stomach tube in the practice of general medicine. By JOHN A. LICHTY, M. D., Clifton Springs.

Discussed by Dr. Benedict, of Buffalo, and then by Dr. Lichty.

Stricture of the urethra. By J. HENRY DOWD, M. D., Buffalo.

Upon motion, the following papers were read by title and ordered published in the transactions: Cause and prevention of sudden death after fifty, by Sydney A. Dunham, M. D., Buffalo; Late additions to our knowledge of certain parasitic affections of the skin, by Frank J. Thornbury, M. D., Buffalo; A plea for local anesthesia in removal of large abdominal tumors in the aged, with the report of a case, by Henry T. Williams, M. D., Rochester; Removal of the uterus in pelvic disease, by C. C. Frederick, M. D., Buffalo; Hysteria and brain tumors, by Wm. C. Krauss, M. D., Buffalo; The ocular headache as an American disease, by Lucien Howe, M. D., Buffalo; The relation of HCL. secretion to indicanuria, by Allen Jones, M. D., Buffalo; Auto-intoxication, by Wm. D. Wolff, M. D., Rochester; Certain cardiac affections in relation to life insurance, by John H. Pryor, M. D., Buffalo; Overdressing of children, by W. M. Brown, M. D., Rochester; A report of a case of puerperal diphtheria, involving the vagina and endometrium, by S. L. Elsner, M. D., Rochester.

The President: Is there any further business before the association?

Dr. YOUNG then offered this motion, which was carried: "In view of the most elegant entertainment we have had here today it is very proper that we offer a vote of thanks to our Buffalo brethren. I move that a vote of thanks be tendered to the Medical Society of the County of Erie for the royal way in which they have entertained the association today."

The PRESIDENT: In closing the meeting for the day I personally wish to thank all those who have participated in the meeting for the thorough work that has been accomplished, for the willingness that has been shown by those who have not been able to complete their papers within the prescribed time to give way to others, but above all, to the local committee of arrangements whereby we have had a most successful meeting brought to a termination. I now declare the thirtieth annual meeting adjourned to meet at Auburn the third Tuesday of October, 1898.

